# Transforming Postpartum Care: The Efficacy of Simulation Training in Hemorrhage Management Among Nurses

**DOI:** 10.3390/healthcare13050549

**Published:** 2025-03-04

**Authors:** Wedad M. Almutairi, Salma M. Almutaraiy, Ahlam Al-Zahrani, Fatmah Alsharif, Wafaa A. Faheem, Areej Abunar, Hala Ahmed Thabet

**Affiliations:** 1 Maternity and Child Department, Faculty of Nursing, King Abdulaziz University, Jeddah 21551, Saudi Arabia; doctor_salma19@hotmail.com (S.M.A.); aealzahrani@kau.edu.sa (A.A.-Z.); wfaheem@kau.edu.sa (W.A.F.); aabunaar@kau.edu.sa (A.A.); hamohammed@kau.edu.sa (H.A.T.); 2 Nursing Services Administration, Ar-Rass General Hospital, Qassim Health Cluster, The Saudi Ministry of Health (MOH), Ar Rass 58883, Saudi Arabia; 3 Medical Surgical Nursing, Faculty of Nursing, King Abdulaziz University, Jeddah 21551, Saudi Arabia; falsharif@kau.edu.sa; 4 Faculty of Nursing, Mansura University, Mansura 35516, Egypt

**Keywords:** postpartum hemorrhage (PPH), prevention, knowledge level, performance, simulation training

## Abstract

**Background/Objectives:** Postpartum hemorrhage (PPH) is the most prevalent complication of childbirth and the most preventable cause of maternal mortality worldwide. Maternity nurses and midwives are often the first-line providers responding to PPH. As a result, maternity nurses have the potential to save the lives of women who are clinically deteriorating because of PPH. Simulation-based training is an effective way to develop maternity nurses’ knowledge, skills, and experience to save a woman’s life after PPH. **Aim:** to investigate the effect of simulation-based training on nurses’ knowledge and performance about primary postpartum hemorrhage management. Design: an experimental design (pre-test/post-test control group). **Setting**: the study was conducted in the labor and delivery room at KAUH in Jeddah, Saudi Arabia. **Sample**: A convenient sample of 54 nurses and midwives who were working in the labor and delivery room and the postnatal unit was randomly divided into two equal groups, the control group and study group. Tools: A structured tool was used for data collection and consisted of four parts: I—sociodemographic data, II—assessment of nurse’s/midwives’ knowledge about prevention and management of primary PPH, III—nurse’s/midwives’ performance observational checklist for primary PPH management, and IV—nurse’s/midwife’s satisfaction of the simulation-based training session. **Results:** The study group had a significantly higher knowledge level immediately after training (X^2^ = 9.39, *p* = 0.002) and one month after training (X^2^ = 5.51, *p* = 0.02). Regarding the performance level and total practice level immediately after the intervention, the study group had statistically significantly better practices (X^2^ = 50.143, *p* = 0.000 *) and this continued one month later (X^2^ = 50.143, *p* = 0.000 *). **Conclusions**: The nurses’ knowledge and performance skills related to primary postpartum hemorrhage care improved after receiving simulation-based training. We recommend that all the maternity nurses and midwives participate in an ongoing in-service simulation training program to enable nurses to demonstrate an active role in PPH prevention and management.

## 1. Introduction

In 2017, 810 women died every day from pregnancy- and birth-related causes that may have been avoided [1]. In 1990, the MMR in the Kingdom of Saudi Arabia (KSA) was 40 per 100,000 live births; however, between 1983 and 2002, it was reduced to 28.4 per 100,000 live births [2]. According to Saudi Arabia Sustainable Development Goal 3 (“Good Health and Well-being: Ensure healthy lives and promote well-being for all at all ages”), the first objective was “By 2030, reduce the global MMR to less than 70 per 100,000 live births” [3]. Notably, the MMR has reduced in recent years: in 2018, the MMR was 11.9 per 100,000 live births, and in 2019, the MMR was 12.0 [4].

Obstetric hemorrhage is the leading cause of maternal mortality (MM) and is responsible for 35.4% of all maternal deaths worldwide [5]. Postpartum hemorrhage (PPH) is the most common complication of childbirth and is the most preventable cause of MM worldwide [6]. According to the WHO, PPH is the main cause of maternal morbidity and mortality globally. PPH is responsible for 35% of maternal fatalities worldwide [7]. PPH is the fifth most common cause of MM, causing 140,000 deaths each year worldwide [8].

The Saudi Ministry of Health (MOH) recorded 9186 incidents of PPH in MOH hospitals in 2017 [9], 8955 PPH incidents in 2018 [10], and 5346 incidents of PPH in 2019 Recently, in 2020, the MOH recorded 5852 out of 235,448 live births [11]. A study in Saudi Arabia found that out of 15,483 deliveries reported in the hospital database, atonic PPH occurred in 386 (2.5%). There were 220 (57%) vaginal births and 166 (43%) cesarean section births among the cases of atonic PPH [12].

Postpartum hemorrhage (PPH) is a life-threatening obstetric emergency that can occur during both vaginal (2–4%) and c-section births (6–7%) because of the third stage of labor [13]. It is a substantial cause of maternal morbidity and one of the top three causes of MM in both high and low-income nations. It is responsible for nearly one-third of all MM globally, with the majority of deaths due to PPH occurring in low-income countries [14].

Postpartum hemorrhage (PH) is defined as the American College of Obstetricians and Gynaecologists (ACOG) as blood loss of more than 500 mL during vaginal birth and more than 1000 mL during cesarean delivery [15]. PPH is often classified as primary/immediate/early, occurring within 24 h of birth, or secondary/delayed/late, occurring more than 24 h after birth to up to 12 weeks postpartum [16]. Uterine atony (impaired uterine contraction after birth) is the most common cause of PPH, contributing to approximately 80% of cases [17].

In atonic PPH, uterotonic drugs such as oxytocin and prostaglandins should be considered as first-line treatments. Second-line treatment includes intrauterine balloon tamponade/invasive therapy, which helps avoid intentional surgery. However, if the second-line treatment fails, surgical procedures like uterine compressive sutures, vascular ligation, and arterial embolization can be considered [18].

In PPH prevention, AMTSL (active management of third stage of labor) is the prophylactic technique that involves the use of uterotonic immediately after delivery of the fetus, controlled cord traction, and fundal massage immediately after delivery of the placenta and palpation of the uterus every 15 min for 2 h to determine the continued need for massage [19]. The administration of a uterotonic drug within one minute after the baby’s birth is part of the AMTSL. Controlled cord traction and uterine tone verification, as well as uterine massage if the uterus is not sufficiently contracted, may be included [20].

Labor and postpartum nurses play a pivotal role in the prevention, early recognition, and management of PPH, especially in the third stage of labor and the early postpartum period, through proper management of the third stage and careful observation of the mother in the early postpartum period. The nurses’ knowledge and training skills were one of the factors that influenced the management of PPH [21]. Insufficient nurses’ knowledge and skills in managing PPH exist [22]. Furthermore, in Saudi Arabia, a majority of nurses are recruited from countries all over the world (e.g., the Philippines, India, South Africa, Malaysia, Europe, and other Arab countries) which impact their practice. To emphasize the nurses’ knowledge and skills in managing PPH, a nursing continued education program should be provided regularly for nurses working in maternity units for any obstetric emergencies.

Simulation is widely utilized in training, and it is gaining popularity in the field of healthcare education. In hospital settings, simulated experiences allow healthcare providers to participate in realistic patient care encounters to which they would not otherwise have access to in real clinical settings. Shoulder dystocia, cord prolapse, PPH, and uterine inversion in the labor and delivery unit are examples of low-frequency or high-impact cases or competencies [23].

Simulation teaching has been defined as “[a] technique to replace or magnify real experiences with guided encounters that evoke or mimic large features of the real world in a fully interactive manner” [24]. Simulation teaching is a viable option for providing education that bridges the gap between theory and practice while reducing patient risk. Acquisition of technical skills and knowledge through simulation leads to an improvement in real-life clinical practice [25]. Furthermore, simulation enables the simultaneous evaluation of both the individual and the full team. In this way, weaknesses in patient management can be highlighted and corrected [24].

Simulation-based training (SBT) has achieved impressive positive results that are reflected in the individuals, the work environment, and the final outputs reached. As the simulation prepares the trainee to integrate into the work, the trainee becomes more powerful and experienced, more able to act correctly, and less likely to make mistakes, thus reducing the chance of failure and increasing the chance of success when working on the ground, thereby achieving the highest success rates [26]. Therefore, a fidelity simulation offers a practical environment with an innovative approach to transferring knowledge into practice. It is critical that maternity nurses engage in ongoing structured simulation training focused on evidence-based nursing practices, as this method is effective in training nurses to act competently in emergencies by focusing on prevention and timely management [27]. Also, simulated training was found to be beneficial in the training of nurses and midwives in the management of obstetric emergencies they experienced in clinical practice. Simulated training has helped participants gain knowledge and confidence as well as retain their knowledge and skills competency for a longer period. Because nurses come from a variety of backgrounds, their skills, experience, and qualifications are highly variable; in addition, because nurses are expected to perform expanded clinical roles, SBT can be a good option for healthcare educators to upskill their nursing staff in competent clinical skills [28]. Since participants practice in a safe, controlled environment during the SBT, which includes PPH situations, such training has been correlated to a higher safety culture and better obstetric outcomes [29].

The prevention and management of PPH are considered vital steps for improving women’s health during childbirth. As a result, maternity nurses and obstetricians must engage in continual simulation training to maintain up-to-date knowledge and professional competency skills and make accurate, precise clinical judgments based on a standard guideline-based methodology for early warning and critical nursing treatment [27]. However, rare studies have been found to investigate the role of SBT in the management of PPH; thus, our study aims to evaluate the effect of SBT on nurses’ knowledge and performance regarding management of the primary PPH management.

The hypothesis for the study is: nurses who have attended SBT about primary PPH management will show a higher knowledge level than those who did not attend such training and nurses who have attended SBT about primary PPH management will reveal better performances than those who did not attend this training.

## 2. Materials and Methods

The design of the study is a pre-test-post-test/control experimental design. Setting: The study was conducted between August 2021 and February 2022 at King Abdulaziz University Hospital’s (KAUH) labor and delivery room (KAUH, the main teaching hospital in Jeddah City, has been in operation). A convenience sample was used for data collection. A total of 54 nurses and midwives working in the labor and delivery room and postnatal unit at KAUH were included in the study.

This study intended to use a convenient sample of all the sixty-six nurses and midwives from the delivery room and postnatal unit. However, 3 of the population was excluded because they had attended simulation courses before the current study, 2 nurses were on their annual leave sometime during the study, 1 senior nurse was dismissed from clinical to record work due to her back injury, and 6 nurses/midwives were included in the pilot study. So, the study sample included 54 nurses/midwives of different ages, years of experience, and qualifications who provide direct nursing care for women in the labor and delivery room and postnatal units at KAUH. Nurses and midwives were selected according to the inclusion criteria and assigned randomly (to avoid sampling bias) into two equal groups (27 participants each in the control and study groups). All nurses/midwives currently employed in the delivery room and postnatal unit at KAUH at the time of data collection were eligible for inclusion. Participants did not need to have managed PPH in the past. Nurses or midwives who were on leave at the time of the study, nurses/midwives who were unwilling to participate in the study or absent in stimulation sessions, and those who had participated in simulation training about PPH or obstetric emergencies at any time before the study were excluded.

The randomization process was conducted using a computer-generated random number table to verify objectivity in selection. An independent investigator, who was not involved in the study’s design or implementation, supervised this process. Baseline demographic data, including age, years of clinical experience, educational background, and exposure to obstetric emergencies, were collected for all participants before randomization. These data were analyzed to confirm that the randomization yielded balanced groups, with no statistically significant differences in these characteristics between the control and study groups. This approach ensured the comparability of groups and strengthened the validity of the study outcomes.

Ethical approval from the Ethical Committee of the Nursing Faculty at KAU in Jeddah (NREC Serial No: Ref No 2M. 74) and from KAUH’s biomedical ethical committee (Reference No. 328-21) and the National Committee of Bio. & Med. Ethics (HA-02-J-008) was obtained. To make it easier for participants to get in touch with them and gather the information needed while maintaining confidentiality and protecting the privacy of their records and information, CSSC-KAU also gave their approval (Reference No. 2m. 74). The researcher obtained written informed permission from each participant after they freely decided to participate, confirming that they understood the study’s purpose and that they were aware that they could withdraw at any time. All the participants’ personal information was kept confidential once the data were gathered. All the data were kept in a secure location before being moved to a laptop that was accessible only to the research teams. All filled questionnaires were then shredded.

Study Tool: the researcher collected data using a structured tool developed by the researcher after an extensive review of the related literature. It consisted of four parts: Socio-demographic and professional characteristics, nurses’/midwives’ knowledge about the management of primary PPH, nurses’/midwives’ performance observational checklist about primary PPH management, and nurses’/midwives’ satisfaction with the SBT session. The researcher used the tool to collect the necessary data from nurses/midwives to address the aim of the study.

The tool includes Part I: Socio-demographic and professional characteristics, which were used to assess the general characteristics of nurses/midwives participating in this study. It included questions about age, nationality, professional qualifications, working unit, years of experience, number of courses for obstetric emergencies attended and whether they have dealt with PPH cases in a real situation. Part II: The nurses’/midwives’ knowledge about PPH management to assess the nurses’/midwives’ knowledge regarding primary PPH management. A multiple-choice questionnaire consisting of 21 questions divided into three main groups was used. The first group of questions assessed general knowledge about primary PPH, including the definition, causes, risk factors, signs, and symptoms (9 questions). The second group of questions included questions about the prevention of primary PPH with AMTSL (5 questions). The third group of questions was about the management of primary PPH (7 questions). This part was scored as follows: A score of 1 was given for correct answers and a score of 0 was given for incorrect/do not know answers. The total possible knowledge score ranged from 0 to 21. A score of ≥11 was considered to indicate good knowledge, while scores < 10 were considered to indicate poor knowledge level. Part III: The nurses’/midwives’ performance observational checklist to assess the nurses’/midwives’ performance skills in managing primary PPH, which include performing the skills in the sequence required (recognition, communication, resuscitation, determining the cause of bleeding, management, and documentation; 18 items). This part was scored as follows: A score of 1 was given if a skill was performed correctly and a score of 0 was given if it was not performed correctly. The possible scores for performance skills ranged from 0 to 18. A score of ≥80% was considered a good performance, while scores < 80% were considered to indicate poor practice.

Instrument validity: To revise the material, three jurors from the fields of obstetric and gynecological nursing and obstetric medicine reviewed the tool’s validity. To fulfill the study’s goal, the questionnaire was adjusted based on their expert comments on sentence clarity, comprehensiveness, and appropriateness of information. A pilot study was also conducted to assess the clarity and applicability of the study tool, as well as the time required to complete the questionnaire. A tenth (10%) of the total sample was used for the pilot study.

Instrument reliability: Statistical tests were used to determine the questionnaire’s internal consistency. Cronbach’s alpha coefficient supported the reliability of the tool employed in this study. The techniques for assessing variables should be dependable to demonstrate the questionnaire’s stability and consistency. In this study, the technique used was found to be dependable, as it produced consistent findings each time the element was measured. In the current study, Cronbach’s alpha for the first domain (knowledge level) was 0.690, which was adequate but not significant. The jury re-examined the knowledge level questionnaire but decided against making any changes as the reduced dependability could be attributable to the small sample size. In contrast, the reliability for the second domain (performing skills), as indicated by Cronbach’s alpha, was 0.915. This result is regarded as excellent, indicating that the instrument can be trusted to measure the study’s objectives.

Intervention: A SBT program was developed by the researcher based on the WHO guidelines for managing complications in pregnancy and childbirth and its updates after a thorough review of the local protocols from KAUH and the local nursing competency in PPH management. The researcher also developed the simulation scenarios based on the WHO Simulation in Nursing and Midwifery Education [20]. Content validity was confirmed by a simulation expert from CSSC. The program objectives were to enhance the participants’ knowledge of primary PPH prevention and management, help participants perform atonic PPH management, and help participants learn how to communicate effectively and appropriately in an emergency. The SBT program had two primary components: (a) A theoretical part: designed in accordance with the training program’s objectives, as well as the related literature, and general knowledge about PPH (definition, etiology, risk factors, signs and symptoms, classification [primary and secondary PPH], recognition and diagnosis, complications, AMTSL, nurse management and evidence-based guidelines for prevention and treatment of PPH) was covered in the theoretical section. The researcher only offered it to the study group.

Assessment phase: Before the training program was implemented, the participants were asked to complete a clinical skills procedure for the management of atonic PPH, which was assessed and filled out by the researcher utilizing part III of the tool. The steps took approximately 10 min for each participant to complete.

The implementation phase (90 min): an SBT program with two main sections (theoretical and clinical) was conducted in situ in the KAUH labor and delivery room. Four sessions were used to implement the curriculum (one session for the theoretical part, one session for the clinical part, one session for the simulation scenario and the last session for debriefing). Theoretical part: A tutorial about prevention and management of PPH (30 min) was offered by the researcher using a PowerPoint presentation. It provided the participants with basic knowledge about primary PPH (definition, causes, risk factors, signs and symptoms, classification, early recognition and diagnosis, complications, nursing management, evidence-based guidelines for prevention and treatment of PPH, and AMTSL for the prevention of PPH). Clinical part: this part was about nursing prevention and management of atonic PPH, the researcher’s demonstration of the nursing assessment for early detection of primary PPH (assessment of history, risk factors and selected laboratory studies that may predispose to PPH), and the clinical/physical examination (uterine assessment) used to confirm signs and symptoms of PPH using a high-fidelity SimMom simulator, an advanced full-body birthing simulator which prepared learners to recognize and respond to high-risk births such as PPH. The simulation scenario part: after completing the skill practice, the researcher ran a scenario of atonic PPH using a high-fidelity SimMom simulator managed by the participants themselves without researcher instructions. Communication with the SimMom simulator was performed by a technician from CSSC-KAU who was not involved in the training sessions. Finally, the debriefing part: Immediately after the simulation, the researcher conducted debriefing as a reflection activity by asking the participants the following reflective questions: How was your experience participating in the virtual scenario? What are your feelings about your performance? What are your feelings on the group’s performance? What went particularly well? Is there anything that could have been done differently? The discussion then proceeded with comments, a review of the protocols and performance during the actual setting, and any unanswered questions.

A pilot study used 10% of the samples, or 6 labor and delivery room and postnatal unit nurses/midwives: 3 nurses/midwives in each subgroup (control and study) who met the inclusion criteria from the chosen setting. The purpose of the pilot study was to determine the tool’s clarity and applicability, as well as how long it would take to complete. To verify the validity of the results, the pilot study participants were eliminated from the research sample. The pilot study was carried out to ensure the validity and reliability of the questionnaire and observational checklist. The researcher determined that no major adjustments to the instrument were required, and no changes were made to the tool after the pilot study. To ensure the validity of the study, all the pilot study participants were not included in the study sample.

Analysis: descriptive statistics were used for categorical variables including frequencies and percentages, continues variables, the mean, the median, and the SD. Chi-squared tests were used to compare the two groups for categorical outcome. The statistical software program Statistical Package for the Social Sciences (SPSS) version 26 was used; significance was set at *p* ≤ 0.05 for all tests. Finally, a comparison between the intervention and control groups was performed to identify the effects of SBT on nurses’ knowledge and performance regarding primary PPH management at KAUH.

## 3. Results

Table 1 displays the distribution of the studied groups according to demographic data. The table shows that more than half of the control and study groups (66.7% and 51.9%, respectively) were aged between 30 and 40 years old, and only 11.1% of the control group and 7.4% of the intervention group were more than 50 years old, with no statistically significant difference in any age group between the intervention and control group; *p* = 0.324. Regarding the nurses’ professional qualifications, less than one-quarter (22.2%) of the control group and 11.1% of the intervention group had a diploma in midwifery, while nearly half of the control and intervention group (40.7% and 59.3%, respectively) had a bachelor’s degree. Furthermore, more than one-third (37.0% and 37.0%) of both groups had 6–10 working years of experience followed by nearly one-quarter (22.2% and 25.9%, respectively) of the control and study groups having 11–15 working years of experience. There were no statistically significant differences between the intervention and control group concerning their professional qualification and years of experience (*p* = 0.342, *p* = 0.637, respectively).

Table 2 illustrates the distribution of the studied groups according to level of knowledge about postpartum hemorrhage before the intervention. Regarding the nurses general knowledge about PPH, nearly three-quarters (74.1%) of both groups had a good general knowledge level. Regarding the prevention of PPH, it was found that the majority (70.4% and 81.5, respectively) of the control and study group had a good level of knowledge.

For the nurses’ knowledge related to the management of PPH, it was found that more than half (63.0%) of the control and more than three-quarters (77.8%) of the study group had a good level of knowledge with no statistically significant differences in the prevention and management of PPH among the control and study group; *p *= 0.340 and 0.233, respectively. In addition, the table displayed that more than half (63.0%) of both groups had a good level in their total knowledge.

Table 3 displayed the distribution of the studied groups according to the level of knowledge about postpartum hemorrhage immediately after the intervention. Regarding the nurse’s general knowledge of PPH, more than three-quarters (77.8%) of the control group as compared to all (100%) of the study group had a good general knowledge level, with a statistically significant difference between the control and the study group regarding the general knowledge about postpartum hemorrhage immediately after the intervention whereas (X^2^ = 6.750, *p *= 0.009 *).

Regarding the prevention of PPH, it was found that nearly three-quarters (70.4%) of the control group as compared to all (100%) of the study group had good knowledge levels with a statistically significant difference between the control and the study group about the knowledge about the prevention of postpartum hemorrhage immediately after the intervention whereas (X^2^ = 9.391, *p *= 0.002 *).

For the nurses’ knowledge related to the management of PPH, it was found that nearly three-quarters (70.4%) of the control group as compared by all (100%) of the study group had a good knowledge level with a statistically significant difference between the control and the study group concerning the knowledge about the management of postpartum hemorrhage immediately after the intervention whereas (X^2^ = 9.391, *p *= 0.002 *). Furthermore, regarding the nurses’ total knowledge, nearly three-quarters (70.4%) of the control group as compared to all (100%) of the study group had good knowledge levels with a statistically significant difference between the control and the study group concerning the total knowledge level about the post-partum hemorrhage immediately after the intervention whereas (X^2^ = 9.391, *p *= 0.002 *).

Table 4 shows the distribution of the studied groups according to knowledge about postpartum hemorrhage after the intervention in the follow-up phase. Regarding the nurse’s general knowledge about PPH, the majority (81.5%) of the control group as compared by all (100%) of the study group had a good general knowledge level with a statistically significant difference between the control and the study group concerning the general knowledge about post-partum hemorrhage after the intervention in the follow-up phase whereas (X^2^ = 5.510, *p *= 0.009 *).

Concerning the prevention of PPH, it was found that the majority (81.5%) of the control group as compared to all (100%) of the study group had a good knowledge level, with a statistically significant difference between the control and the study group regarding the knowledge about the prevention of postpartum hemorrhage after intervention in the follow-up phase, with (X^2^ = 5.510, *p *= 0.009 *).

For the nurses’ knowledge related to the management of PPH, it was found that the majority (88.9%) of the control group when compared to all (100%) of the study group had good knowledge levels with no statistically significant difference between the control and the study group regarding knowledge about the management of postpartum hemorrhage after intervention in the follow-up phase, with (X^2^ = 3.176, *p *= 0.075).

Furthermore, regarding the nurse’s total knowledge, the majority (81.5%) of the control group when compared to all (100%) of the study group had good knowledge levels, with a statistically significant difference between the control and the study group regarding the total knowledge level of post-partum hemorrhage after intervention in the follow-up phase, with (X^2^ = 5.510, *p *= 0.009 *).

## 4. Discussion

The current study aimed to evaluate the effect of SBT on maternity nurses’ knowledge and performance skills regarding primary PPH management at King Abdulaziz University Hospital in Jeddah, Saudi Arabia. Regarding the maternity nurses’ and midwives’ ages, more than half of the participants in this study were more than 30 and less than 40 years old. The present study’s findings are consistent with those of a study previously conducted at Walden University, which also found that the most common age range was 31–40 years (46.7%) [30]. A study conducted by Angelina group showed that 40.7% of the studied nurses were ≥40 years old. Moreover, the findings of [31] revealed that over one-third of the studied nurses were 30 years of age or younger. On the other hand, the findings of the current study disagreed with those of [32], who conducted research in Africa on the knowledge, attitudes, and practices of Ghanaian midwives and found that the most common age range among the participating midwives was from 20 to 30 years old (39.3%). In addition, the findings of [33] contradict those of the current study, as they showed that approximately two-thirds (61.5%) of maternity nurses studied were under 30 years of age.

In addition, in a study conducted in Iraq that assessed nurses’ and midwives’ knowledge regarding the management and prevention of PPH, the plurality of the study’s participants (44.7%) were 20–29 years of age [34]. Moreover, in contrast to the current study, ref. [35] found that half of the nurses in their study were between 41 and 50 years of age.

The range is important because nurses and midwives in this group usually have enough clinical experience to perform well, but they are also willing to learn new skills and adjust to cutting-edge methods like simulation-based training (SBT). Younger nurses (20–29 years old) may have less experience, but they are frequently more willing to learn new things and participate in skill-building activities. A possible explanation for the consistencies (and inconsistencies) between the results of this study and the previous studies discussed is that Vision 2030, which aims to enhance and properly use the capability of health centers and hospitals as well as expand the standards of healthcare services, is not hiring persons over 50 years of age. The study results could also be attributed to the fact that persons aged 30 to 40 years tend to be more skilled and mature than younger individuals, who often try various careers and have a high turnover rate; in addition, persons aged 30 to 40 years are particularly vital, as older individuals are at greater risk of burnout.

Regarding the participants’ level of education, the current study showed that nearly half of all participants in the control and intervention groups had a bachelor’s degree, while one-third of the total sample had a diploma in nursing, and only 16% of the studied group had a midwifery diploma. No studies whose results agreed with those of the current study were found. This study’s findings disagreed with those of the study by Ahmed Elkholy et al. [36] which showed that approximately two-thirds (60%) of studied nurses had nursing diplomas and one-third (32%) had a certificate from a technical institute of midwifery, while only (8%) held a bachelor’s degree in nursing. The current study’s findings also disagreed with those of Ali & Bahaaldeen [37] who found that the majority of study respondents (57.7%) graduated from a technical institute of midwifery (secondary level), while only 3.1% had a bachelor’s degree from a nursing college.

Also in contrast to this study, in a study conducted in Iraq that assessed nurses’ and midwives’ knowledge regarding the management and prevention of PPH, most of the study’s participants (111/150; 74%) had a secondary-level degree in nurse-midwifery. Moreover, a study conducted by Abd Elhakm & Elbana [34] revealed that approximately half (53.8%) of studied maternity nurses had a secondary nursing education.

The discrepancies between the findings of this study and some previous studies could be due in part to the Vision 2030 and National Transformation Program’s approval in June 2016, which includes 15 tactical objectives and 16 Key Performance Indicators (KPIs) for the health segment. One of these KPIs is to improve quality and safety measures, as well as the performance of healthcare providers; thus, the government has made an admirable effort to help Saudi young people join the constructive educational atmospheres at faculties, universities, and sufficiently equipped teaching hospitals. As a result, the majority of hired individuals have a bachelor’s degree or more. Moreover, the Dominion of the Kingdom of Saudi Arabia and the Ministry of Health closed all nursing diploma programs in 2012 and encouraged the previously graduated diploma nurses to bridge their qualifications, giving them all the opportunities and facilities to complete their academic degrees.

Regarding the number of years of experience, the results of the current study revealed that the majority of the participants had less than 10 years of experience in maternity units. The present study results are congruent with those of the study by Abd Elhakm & Elbana [34], which revealed that the majority of the studied sample (86.2%) had less than 10 years of experience. In addition, in keeping with this study’s findings, a study by Medoh [30] revealed that 66.7% of participants had less than 10 years of experience as a labor/delivery, postpartum and/or an obstetric nurse. Similarly analogous to this study’s finding, Ali & Bahaaldeen [37] found that the bulk of their sample had completed less than 10 years of service (93.8%); nearly two-thirds of their entire sample (57.7%) had 1 to 5 years of experience. On the other hand, Ahmed Elkholy et al. [36] found that two-thirds (64%) of the nurses in their study had over 10 years of working experience. A possible explanation for any disagreements between this study’s findings and those of previous studies is that differences in working experience could be due to differences in the studies’ demographics, as the majority of the nurses in the current study were aged 30 to 40 years and most of them had a bachelor’s degree; hence, they have less than 10 years’ experience due to the graduation age and hiring policies.

Nurses’ and midwives’ primary PPH practice levels before the intervention: Regarding the practice related to primary PPH management among maternity nurses and midwives before the intervention, the present study findings revealed that more than three-quarters of the studied maternity nurses had unsatisfactory practice. For PPH in the pre-program period, there were no statistically significant differences between the study and control groups. The present study results were in line with those of the skill examination reported by [31], which showed that only one-third of the participants had adequate skills in PPH management, while nearly two-thirds had inadequate skills. The current study findings were also congruent with the findings of [34], who reported that nurses’ performance in AMTSL was inadequate before participating in a simulation educational program. Similarly, ref. [27] found that a simulation educational program improved the nurses’ performance. The degree of nurses’ performance was unsatisfactory before attending the simulation educational program.

On the other hand, the findings of this study are dissimilar to those of Odesanya et al. [38], who showed that the vast majority of the birth providers in their study (127 out of 128; 99.2%) demonstrated a good practice of prevention of PPH, while 1 participant out of 128 (0.8%) had a poor level of practice, and none had a fair level of practice of prevention of PPH. Thus, primary health workers at the Osobo Center in Osun State, where the cited study was conducted, are skilled delivery providers with a good level of prevention of PPH. The present study results are also in contrast with those of [32], who found that more than one-third of nurses had a satisfactory level of practice for immediate postpartum care for mothers throughout the pre-program application period.

The results of the present study, as well as the results of the other studies mentioned, are reflected in the researcher’s close observation, which revealed a poor level of practice among both groups before the implementation of the training simulation, which included resuscitation, determining the cause of bleeding, PPH management, and documentation. These findings may also be attributed to the fact that, according to the KSA hospital policies and nurses’ job specifications, nurses are accustomed to calling only the doctor on duty, as they are the person responsible in an emergency, and there is a high availability of resident physicians compared to other countries. It can be added that the unacceptable performance skill level among the current study participants may be the result of the absence of emergency obstetric drills.

The effectiveness of the SBT in improving nurses’ knowledge, practice, and satisfaction regarding PPH: Regarding the effect of simulation-based education on the nurses’ knowledge, in the current study, it is hypothesized that nurses who have attended SBT about primary PPH management will show a higher knowledge level than those who did not attend. The results of the present study revealed that before the intervention, there were no statistically significant differences between the control and study groups in general regarding PPH knowledge, knowledge about prevention of PPH, knowledge about management of PPH, or total knowledge about PPH. In contrast, there was a statistically significant difference between the control and study groups in these measures immediately after the intervention and in the follow-up phase. This means that nurses who attended SBT on the management of primary PPH demonstrated a higher level of knowledge about PPH management than those who did not. The results of the present study are consistent with the results of the study conducted by Nelissen [39], which indicated that there was a major improvement in knowledge and abilities related to PPH after an educational program was introduced. The results of the current study were also confirmed by studies conducted by Abd Elhakm and Elbana [34]. The former established a significant improvement in knowledge levels between the study groups after one and three months of simulation training, while the latter indicated that the nurses’ knowledge improved dramatically after simulation training for PPH. Furthermore, in a study by Meza et al. [40], SBT was proven to be a good and feasible technique to boost short- and long-term clinical knowledge of obstetric emergencies. Likewise, a study conducted by El-Hamid [7] showed that the students’ knowledge of PPH was statistically significantly higher after participating in a high-fidelity simulation-based educational program than after receiving traditional clinical training (*p* = 0.001). Consistent with the present study results, Angelina [31] highlighted that a simulated intervention resulted in a positive change in nurses’ knowledge and competencies in AMTSL shortly after the intervention.

In agreement with the results of this study, ref. [39] found that students had increased knowledge about PPH, and their ability to make the proper decision about when to inform the physician strengthened after simulation training. A similar study was conducted by Nishimwe et al. to evaluate the impact of the M-teaching application on the nurses’ and midwives’ knowledge and skills in PPH management and neonatal resuscitation in two regional hospitals in Rwanda. They found a statistically significant upsurge in the mean knowledge scores after the intervention, demonstrating a 17.1% increase in the mean score for PPH management [41].

A study conducted by Kumar et al. matched simulation-based instruction with instructive lectures and detected that simulation-based instruction is a better strategy for teaching/learning than instructive lectures, particularly for obstetric emergencies. An evaluation of grades on a post-lecture multiple choice quiz showed that both groups revealed significant improvement compared with performances on a pre-lecture multiple choice quiz, with more enhancement detected among the simulation group (mean ± SD 18.60 ± 1.90) [42]. The results of the current study follow those of a study conducted by Nelissen et al. which revealed that the inclusion of obstetric SBT was accompanied by a reduction in the occurrence of PPH of over one-third and an improved clinical performance of fundamental delivery skills and PPH care [39]. They are also in line with the results of the study by [33], which found that SBT improved nurses’ performance (knowledge and practice) and self-confidence in primary PPH care. Furthermore, the findings of Lutgendorf demonstrated that simulation training helped boost procedures, team performance, operational readiness, and system improvements. Simulation-based midwifery training improved the Ethiopian staff’s teaching skills and increased their ability to deal with a variety of adverse obstetric situations [43].

Consistent with the current study results, ref. [14] found that medical students who received simulation training on vaginal examinations had more confidence in performing vaginal examinations than the control group. Moreover, the experimental group’s practice level was significantly higher than the control group of students who had not performed any simulator training procedures. Likewise, a study conducted by [17] revealed that there were highly statistically significant differences (*p* < 0.001) between nurses’ pre- and post-intervention performance of peripheral pulse assessment; blood pressure assessment; fundus, lochia, and perineal assessment; urinary catheterization; administration of ecbolic drugs and intravenous fluid; uterine massage; and perineal precaution and total practice scores.

The results of the current study and those with similar findings may be due to the efficacious and efficient use of SBT, which permits the nurses to participate in simulated environments using relevant and realistic PPH scenarios and helps them increase their practice level. The results may also be since simulation is an efficient way to induce knowledge retainment and skill improvement that affects learners’ cognitive and psychomotor domains by moving the learner beyond memorization to actual application and understanding. In addition, SBT is a highly efficacious method of transferring key skills to trainees in an exceedingly safe, comfortable manner. It provides an optimal way for trainers to assess how well their trainees are utilizing their skills in practice and the decisions they are making in simulated reality situations.

The limitations: Performing the study in one setting with a small sample size imitated the generalizability of our findings even though we have heterogeneity of participants. Moreover, the researchers faced challenges when scheduling the session due to the nurses’ shifts changes, which lead to prolongation of data collection. 

Recommendations: There is a need for a major national study to examine the nurses’ and midwives’ knowledge and skills, as well as an interventional study to address knowledge and skill gaps. In an ideal situation, such studies would focus on pre-service and in-service nurses and midwives receiving novel obstetric life-saving skills training.

To improve the generalizability of the findings, the study should be replicated with a larger sample size and in various health institutions in Saudi Arabia. It is also recommended that this study be carried out with more varied sample groups (obstetricians, emergency physicians, nurses, etc.), and the impact of simulation training on PPH prevention and management in other dimensions should be examined, such as maternal and fetal outcomes and follow-up studies.

## 5. Conclusions

The findings of this study provide undeniable proof of the benefits of simulation training in improving and maintaining nurses’ and midwives’ knowledge and performance skills in all areas of PPH management (assessment, resuscitation, arresting bleeding, monitoring and investigation, and evaluation and documentation) in the study group compared to the control group immediately and one month of conducting the simulation training program. In contrast to the study group after the SBT, the control group reported the same level of knowledge and inadequate performance skills.

There was a statistically significant difference in the knowledge and performance skills of the nurses and midwives before and after the program was conducted. Nurses must be evaluated and educated regularly to identify and correct any deficiencies in their knowledge and skills. After SBT, the mean scores of knowledge, clinical skills, team collaboration, and learning satisfaction were close to the maximum score. In conclusion, PPH remains a leading cause of maternal death. For this reason, prevention and proper treatment are critical. However, the study’s outcomes are in line with earlier findings showing that an education program with simulation training can increase the clinical understanding of primary PPH management, clinical skills, teamwork, and communication during an emergency.

## Figures and Tables

**Table 1:** Frequency distribution of the studied groups according to demographic data.

Demographic Data		Groups				Test of Significance
		Control (n = 27)		Study (n = 27)		
		No.	%	No.	%	
Age (years)	20-	2	7.4	7	25.9	X^2^ = 3.478 *p *= 0.324
	30-	18	66.7	14	51.9	
	40-	4	14.8	4	14.8	
	≥50	3	11.1	2	7.4	
Nationality	Saudi	4	14.8	7	25.9	X^2^ = 1.027 *p *= 0.311
	Non-Saudi	23	85.2	20	74.1	
Working unit	Delivery room	11	40.7	15	55.6	X^2^ = 1.187 *p *= 0.276
	Post-natal unit	16	59.3	12	44.4	
Professional qualification	Diploma of midwifery	6	22.2	3	11.1	X^2^ = 2.148 *p *= 0.342
	Diploma of nursing	10	37.0	8	29.6	
	Bachelor’s degree	11	40.7	16	59.3	
Years of experience	<2	2	7.4	0	0.0	X^2^ = 3.410 *p *= 0.637
	2–5	6	22.2	6	22.2	
	6–10	10	37.0	10	37.0	
	11–15	6	22.2	7	25.9	
	16–20	2	7.4	1	3.7	
	>20	1	3.7	3	11.1	
Number of training courses	None	5	18.5	7	25.9	X^2^ = 2.005 *p *= 0.571
	1–2	17	63.0	12	44.4	
	3–4	3	11.1	4	14.8	
	≥5	2	7.4	4	14.8	
Managed PPH in real situation	Yes	22	81.5	18	66.7	X^2^ = 1.543 *p *= 0.214
	No	5	18.5	9	33.3	
Participated in simulation training	Yes	0	0.0	0	0.0	X^2^ = ND
	No	27	100.0	27	100.0	

X^2^ Chi-squared test, ND = no differences.

**Table 2:** Frequency distribution of the studied groups according to level of knowledge postpartum hemorrhage before the intervention.

Items		Groups				Test of Significance
		Control (n = 27)		Study (n = 27)		
		No.	%	No.	%	
General knowledge about PPH	Poor	7	25.9	7	25.9	X^2^ = NA
	Good	20	74.1	20	74.1	
Prevention of PPH	Poor	8	29.6	5	18.5	X^2^ = 0.912 *p *= 0.340
	Good	19	70.4	22	81.5	
Management of PPH	Poor	10	37.0	6	22.2	X^2^ = 1.421 *p *= 0.233
	Good	17	63.0	21	77.8	
Total nurses’ knowledge about PPH	Poor	10	37.0	10	37.0	X^2^ = NA
	Good	17	63.0	17	63.0	

X^2^ Chi-squared test, NA = not applicable.

**Table 3:** Frequency distribution of the studied groups according to level of knowledge postpartum hemorrhage immediately the intervention.

Items		Groups				Test of Significance
		Control (n = 27)		Study (n = 27)		
		No.	%	No.	%	
General knowledge about PPH	Poor	6	22.2	0	0.0	X^2^ = 6.750 *p *= 0.009 *
	Good	21	77.8	27	100.0	
Prevention of PPH	Poor	8	29.6	0	0.0	X^2^ = 9.391 *p *= 0.002 *
	Good	19	70.4	27	100.0	
Management of PPH	Poor	8	29.6	0	0.0	X^2^ = 9.391 *p *= 0.002 *
	Good	19	70.4	27	100.0	
Total nurses’ knowledge of PPH	Poor	8	29.6	0	0.0	X^2^ = 9.391 *p *= 0.002 *
	Good	19	70.4	27	100.0	

X^2^ Chi-squared test, * Significant *p* at ≤0.05.

**Table 4:** Frequency distribution of the studied groups according to knowledge about post-partum hemorrhage after the intervention in the follow-up phase.

Items		Groups				Test of Significance
		Control (n = 27)		Study (n = 27)		
		No.	%	No.	%	
General knowledge about PPH	Poor	5	18.5	0	0.0	X^2^ = 5.510 *p *= 0.019 *
	Good	22	81.5	27	100.0	
Prevention of PPH	Poor	5	18.5	0	0.0	X^2^ = 5.510 *p *= 0.019 *
	Good	22	81.5	27	100.0	
Management of PPH	Poor	3	11.1	0	0.0	X^2^ = 3.176 *p *= 0.075
	Good	24	88.9	27	100.0	
Total nurses’ knowledge of PPH	Poor	5	18.5	0	0.0	X^2^ = 5.510 *p *= 0.019 *
	Good	22	81.5	27	100.0	

X^2^ Chi-squared test, * Significant *p* at ≤0.05.

## Data Availability

The original contributions presented in this study are included in the article. Further inquiries can be directed to the corresponding author(s).
